# Inhibition of Lysyl Oxidases Improves Drug Diffusion and Increases Efficacy of Cytotoxic Treatment in 3D Tumor Models

**DOI:** 10.1038/srep17576

**Published:** 2015-12-01

**Authors:** Friedrich Schütze, Florian Röhrig, Sandra Vorlová, Sabine Gätzner, Anja Kuhn, Süleyman Ergün, Erik Henke

**Affiliations:** 1 Institute of Anatomy and Cell Biology II, Universität Würzburg Koellikerstrasse 6, Würzburg, 97070, Germany; 2 Institute of Clinical Biochemistry and Pathobiochemistry, Universitätsklinikum Würzburg Josef-Schneider-Strasse 2, Würzburg, 97080, Germany; 3 Institute of Tissue Engineering, Universität Würzburg Roentgenring 11, Würzburg, 97070, Germany; 4 Graduate School for Life Science, Universität Würzburg Josef-Schneider-Strasse 2, Würzburg, 97080, Germany

## Abstract

Tumors are characterized by a rigid, highly cross-linked extracellular matrix (ECM), which impedes homogeneous drug distribution and potentially protects malignant cells from exposure to therapeutics. Lysyl oxidases are major contributors to tissue stiffness and the elevated expression of these enzymes observed in most cancers might influence drug distribution and efficacy. We examined the effect of lysyl oxidases on drug distribution and efficacy in 3D *in vitro* assay systems. In our experiments elevated lysyl oxidase activity was responsible for reduced drug diffusion under hypoxic conditions and consequently impaired cytotoxicity of various chemotherapeutics. This effect was only observed in 3D settings but not in 2D-cell culture, confirming that lysyl oxidases affect drug efficacy by modification of the ECM and do not confer a direct desensitizing effect. Both drug diffusion and efficacy were strongly enhanced by inhibition of lysyl oxidases. The results from the *in vitro* experiments correlated with tumor drug distribution *in vivo*, and predicted response to therapeutics in murine tumor models. Our results demonstrate that lysyl oxidase activity modulates the physical barrier function of ECM for small molecule drugs influencing their therapeutic efficacy. Targeting this process has the potential to significantly enhance therapeutic efficacy in the treatment of malignant diseases.

Systemic therapy is one of the cornerstones in the management of cancer. It is of particular importance for the treatment of progressed metastatic disease when local therapeutic options like surgery and radiotherapy can only have limited effect. However, systemic treatment is rarely curative and it is estimated that in around 90% of cases chemotherapeutics are administered in a purely palliative setting, aiming to stabilize disease, prolong survival and improve quality of life^1^. There are two major reasons for this situation: first the considerable side effects and the small therapeutic window of most anti-neoplastic drugs, leads in a significant fraction of patients to a premature termination of the treatment. Second, development of resistance to the therapy is ultimately observed in almost all patients. Both of these problems are aggravated by the ineffective transport of systemic drugs into the tumor and to the malignant cells. It has been demonstrated for a variety of different drugs that their concentration after systemic application is significantly lower in the tumor tissue than in non-target organs^2^,^3^,^4^. Moreover, the drug distribution within solid tumors is heterogeneous, leaving large parts of the tumor protected from therapeutically effective drug concentrations^5^. The impaired drug transport necessitates higher dosage of the drugs, leading to higher systemic exposure and subsequently increased risk of side effects. On the other side, exposing tumor cells to low, sub-lethal drug levels as a direct result of the impaired, heterogeneous intratumoral distribution, effectively selects for resistance.

The defective tumor vasculature has been extensively discussed as a main reason for the impaireded tumor drug delivery^6^. The persistently strongly pro-angiogenic milieu in the tumor micro-environment keeps vascular endothelial cells in a constantly activated state, leading to permanent vessel remodeling, deficient support by pericytes and vascular permeability. Various anti-angiogenic strategies have been investigated with the aim to improve drug delivery^7^,^8^,^9^,^10^.

However, to be effective, anti-neoplastic drugs must not only be transported into the tumor, but they also have to reach the tumor cells at a critical concentration. The rate of diffusion within the tissue depends on the characteristics of the extracellular matrix (ECM), which forms a barrier that drugs first have to cross before reaching the individual tumor cells. Like the tumor vasculature, the ECM in tumors differs significantly from the ECM in normal tissue. The most apparent is the abundance of extracellular material in many tumors, with ECM often representing 30–60% of the tumor volume^11^. Also its composition, the ratio of different ECM contributing macromolecules, differs significantly from the ECM in the surrounding tissue of origin^12^,^13^,^14^,^15^,^16^. Finally, matrix modifying, stabilizing and degrading enzymes like metallo proteinases, and lysyl oxidases are strongly dysregulated in cancer^17^,^18^,^19^. The altered biochemical condition also influences the biomechanical characteristics of the pathological tumor microenvironment. Tumor tissue is more rigid than normal tissue, and the physical barrier function of the ECM impeding diffusion is increased, strongly inhibiting transport and efficacy of macromolecular drugs^20^,^21^,^22^,^23^. Experimental data on how ECM characteristics influence transport and efficacy of small molecule drugs however is still limited.

Stability and biomechanical properties of the ECM are strongly influenced by the activity lysyl oxidases, a family of five copper-enzymes (LOX and LOXL1-4), which catalyze the cross-linking of collagens and elastin^24^,^25^. The cross-linking not only stabilizes these proteins, but also increases tissue stiffness and in all probability reduces diffusion through the ECM. Lysyl oxidases have been consistently found to be up-regulated in solid tumors^26^,^27^,^28^. In contrast to the structural components of the ECM, lysyl oxidases as enzymes are well targetable by pharmacological means. Thus, inhibition of lysyl oxidases or modulation of their activity might be an interesting approach to reduce tumor stiffness and potentially improve tumor drug distribution.

To get a better understanding of the influence of ECM crosslinking on drug delivery, the effect of lysyl oxidases on the diffusion and efficacy of small molecular drugs was studied in 3D *in vitro* assay systems. Our results demonstrate that lysyl oxidases activity indeed strongly influences drug diffusion and thereby cytotoxicity of chemotherapeutic drugs. This effect is restricted to 3D assay conditions, while lysyl oxidases do not affect drug efficacy towards tumor cells grown in 2D. Moreover, our results show that the efficacy of various cytotoxic drugs is differently affected by increased lysyl oxidase activity, a finding we were able to corroborate *in vivo*.

## Results

### Increased lysyl oxidase activity reduces drug diffusion

**Figure 1:**
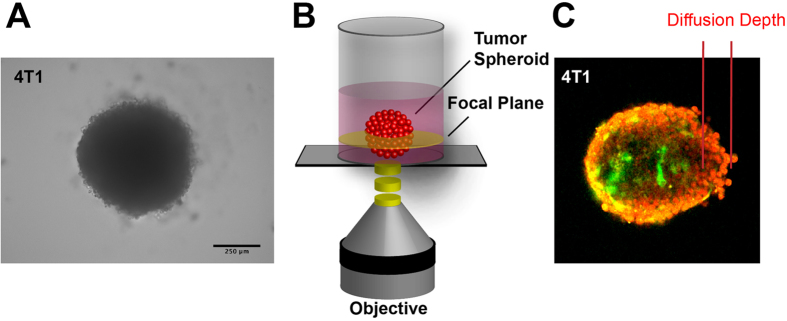
Tumor Spheroid Assay for Determining Drug Diffusion Rates. (**A**) Micrograph of multicellular tumor spheroid generated from 4T1 breast carcinoma cells. Scale Bar: 250 μm. (**B**) Experimental setup. (**C**) Confocal image of tumor spheroid generated from GFP-expressing 4T1 tumor cells after exposure to doxorubicin (red channel). Scale bar: 100 μm.

**Figure 2:**
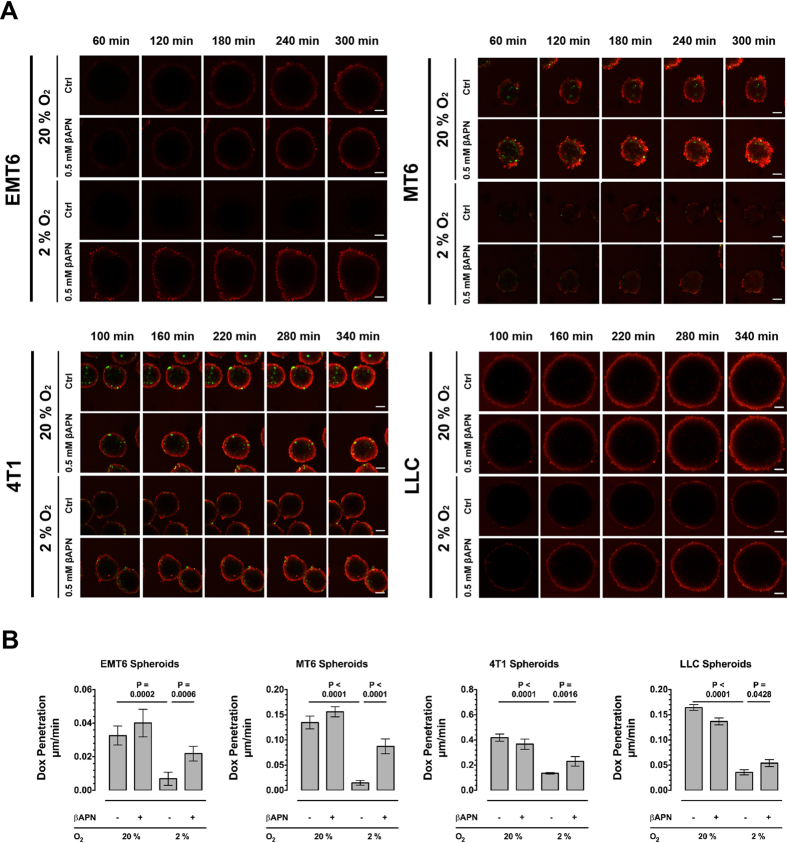
Lysyl Oxidase Activity reduces Drug Diffusion Rate in Multicellular Tumor Spheroids. (**A**) Time course of doxorubicin diffusion in tumor spheroids generated from murine 4T1 breast carcinoma, MT6 fibrosarcoma, EMT6 breast carcinoma and Lewis Lung Carcinoma cells after cultivation at 20% or 2% oxygen. (**B**) Absolute diffusion rates into tumor spheroids. Images acquired on a laser scanning confocal microscope (n = 12), Scale bars: 100 μm, Error bars: ±SEM.

We have shown recently that lysyl oxidase inhibition improved response to cytotoxic drugs *in vivo*. The results indicated that lysyl oxidase catalyzed ECM-crosslinking impedes tumor drug diffusion, thereby forming a physical barrier that protects tumor cells from exposure to the systemically applied drugs. However, *in vivo* matrix changes caused by inhibition of lysyl oxidases also have secondary effects on the tumor microenvironment, on stroma cell populations and on the tumor vasculature^29^,^30^. These effects might also affect drug delivery and efficacy. Consequently, to test the effect of lysyl oxidases on drug diffusion unaffected from these secondary effects, we established a tumor spheroid-based *in vitro* assay system (Fig. 1). Spheroids were generated by culturing tumor cells in absence of a suitable attachment surface, using the liquid overlay technique^31^,^32^. With the compact spheroids formed after six days of culture it was possible to measure the diffusion of fluorescent drugs, like doxorubicin (DOX), via laser scanning confocal microscopy in real time. We used spheroids generated from four different murine tumor cell lines—lewis lung carcinoma (LLC), a fibrosarcoma line (MT6) and two breast carcinoma lines (4T1, EMT6). The diffusion rate differed significantly between spheroids generated from the various lines (Fig. 2A,B). To examine the effect of lysyl oxidase on drug diffusion, we cultivated the spheroids in presence of 500 μM 3-amino propionitrile (βAPN), an inhibitor of all five lysyl oxidase family members^33^,^34^,^35^. Even at this high concentration βAPN did not affect formation or size of the generated spheroids (Supplemental Fig. 1). When tumor spheroids were cultivated at standard levels of 20% O_2,_ βAPN-treatment did not significantly affect diffusion of doxorubicin (DOX). However, when the spheroids were cultivated at reduced oxygen levels (2% O_2_), mimicking hypoxic conditions seen in tumors, DOX diffusion was significantly reduced in the spheres generated from all four cell lines. Under the reduced oxygenation levels βAPN-treatment was able to at least partially restore DOX diffusion to levels observed under high oxygen exposure. To validate that βAPN treatment indeed reduces collagen crosslinking we treated tumor bearing mice for 10 days with βAPN, and evaluated the ECM from the treated tumors by interferences reflection microscopy^36^. βAPN treatment significantly reduced the number of crosslinked colagen fibers (Supplemental Fig. 2).

**Figure 3:**
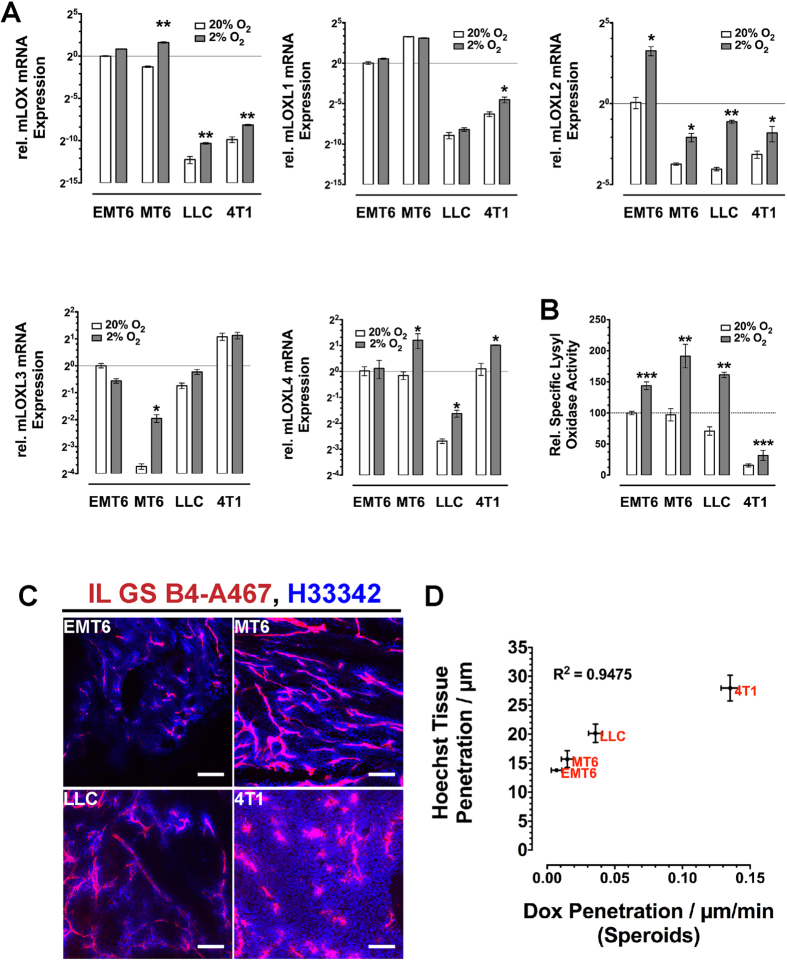
Lysyl Oxidase Expression is Increased Under Reduced Oxygen Supply. (**A**) mRNA expression of the five lysyl oxidase family members in murine tumor cells cultivated at 20% or 2% oxygen levels (n = 3). (**B**) Lysyl oxidase activity in the supernatant of murine tumor cells cultivated at 20% or 2% oxygen levels (n = 4). (**C**) Extravasation of Hoechst 33342 (blue) from vessels (Isolectin GS-B4, red) in implanted tumors. (**D**) Correlation between Hoechst 33342 penetration in tumors and DOX diffusion in tumor spheroids generated from different murine cell lines. Error bars: ±SEM. Scale bars: 100 μm. *indicate statistical significance versus respective controls, *P < 0.05, **P < 0.01, ***P < 0.001 and mRNA levels changed at least 2-fold.

It has been demonstrated that the lysyl oxidase family members LOX, LOXL2 and LOXL4 are regulated by the Hif1α/β transcription factors^28^,^36^,^37^. We analyzed the promoter regions of the LOX proteins for the HIF binding site consensus sequence 5′-(R)CGTG-3′^38^. In the promoter regions of the human genes lox, loxl1 and loxl4, and of the murine genes *lox* and *loxl1* two copies of the consensus sequences as inverted repeats could be found (Supplemental Fig. 3). Thus, cells cultivated under reduced oxygen levels can be expected to secrete elevated levels of lysyl oxidases. However, it has been shown previously that depending on the analyzed cancer cell line, different members of the lysyl oxidase family are up-regulated in response to reduced oxygen supply^36^. We therefore tested the four cell lines in our experiments for changes in the mRNA expression of the five lysyl oxidases under reduced oxygen levels (Fig. 3A). Most consistently mRNA-levels of LOX, LOXL2 and LOXL4 were up-regulated. LOXL2 was stronger expressed in all four cell lines, and also showed the strongest increase in mRNA-levels (9.5-fold in MT6, 7.6-fold in LLC cells). LOX and LOXL4 were up-regulated in all but EMT6 cells. In contrast LOXL1 and LOXL3 were only significantly up-regulated in 4T1 and MT6 cells respectively. The up-regulation of mRNA-levels also resulted in increased overall lysyl oxidase activity in the supernatant of cells cultivated at oxygen levels of 2% (Fig. 3B). In all four lines cultivation at reduced oxygen levels increased secreted lysyl oxidase activity within the supernatant by 43% to 127%.

In the spheroid assay, diffusion rates of DOX differed significantly between spheroids generated from different cell lines. We subsequently tested if this is also reflected in the corresponding tumors generated by implantation of the respective cell lines. Tumor bearing mice were injected with the fluorescent tracer Hoechst 33342 and fluorophor-labeled isolectin GS-B4 and sacrificed 20 min later. In the harvested tumors, diffusivity for small molecule drugs could be estimated by the penetration depth of Hoechst 33342 from the stained vessels into the tumor parenchyma (Fig. 3C). Penetration depth varied significantly in the various tumors and was correlated with the diffusion rates for DOX observed *in vitro* (Fig. 3D).

### Recombinant overexpression of lysyl oxidases reduces drug diffusion

**Figure 4:**
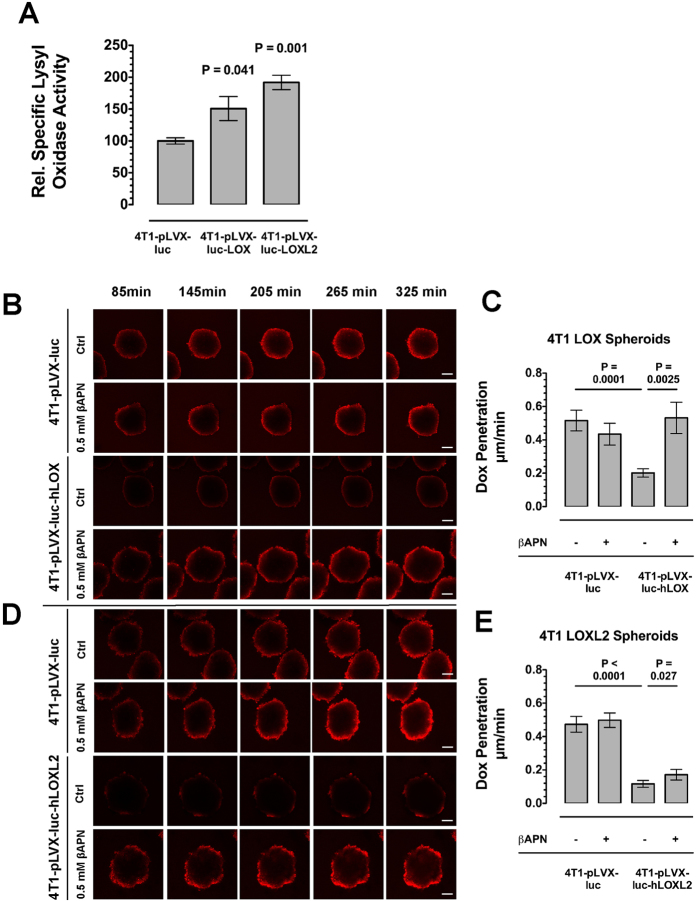
Recombinant Overexpression of Lysyl Oxidases Reduces Drug Diffusion in Tumor Spheroids. (**A**) Relative lysyl oxidase activity in the supernatant of transfected and selected 4T1 cells (n = 4). (**B**) Time course of doxorubicin diffusion in tumor spheroids generated from 4T1 cells overexpressing hLOX versus control transfected cells. (**C**) Absolute diffusion rates into tumor spheroids generated from 4T1 cells overexpressing hLOX. (**D**) Time course of doxorubicin diffusion in tumor spheroids generated from 4T1 cells overexpressing hLOXL2 versus control transfected cells. (**E**) Absolute diffusion rates into tumor spheroids generated from 4T1 cells overexpressing hLOX. Tumor spheroids cultivated at 20% oxygen (n = 12). All images acquired on a laser scanning confocal microscope, Scale bars: 100 μm, Error bars: ±SEM.

To further corroborate the concept that lysyl oxidases directly influence drug diffusity and efficacy, we constructed lentiviral vectors for the constitutive overexpression of hLOX and hLOXL2 in mammalian cells (Supplemental Fig. 4). Lentiviral pseudoparticles generated with these vectors in HEK293T cells were used to infect 4T1 cells, which were selected for these experiments because they displayed the lowest basal expression of lysyl oxidases. We first tested that the recombinant lysyl oxidases were secreted in active form into the culture supernatant (Fig. 4A). Tumor spheroids from control transfected and LOX/LOXL2 overexpressing 4T1 cells were generated and cultivated at 20% oxygen levels to keep interferences by endogenous lysyl oxidases at a minimum. Tumor spheroids generated from the overexpressing cells showed a strongly impaired diffusion of DOX (Fig. 4B,D). The effect was stronger in the hLOXL2 overexpressing 4T1 cells compared to spheroids generated from 4T1 cells expressing hLOX (Fig. 4C,E). Although βAPN significantly improved drug diffusion in the hLOXL2 spheroids, the reduced diffusivity in the 4T1 hLOXL2 spheroids could not completely be restored by incubation with βAPN.

### Impaired drug diffusion after matrix crosslinking affects efficacy of cytotoxic drugs

**Figure 5:**
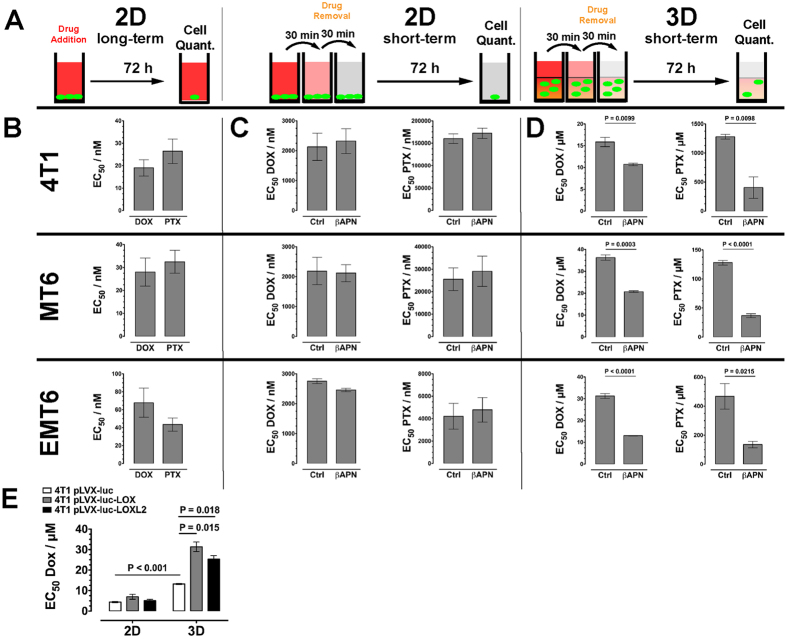
Cell Toxicity of Cytotoxic Drugs in 2D and 3D Culture. (**A**) Experimental setup of drug exposure times and cultivation conditions. (**B**) EC_50_ values for PTX and DOX after exposure of cells cultivated in 2D and exposure to the drugs for 72 h. (**C**) EC_50_ values for PTX and DOX after exposure of cells cultivated in 2D and exposure to the drugs for 30 min. (**D**) EC_50_ values for PTX and DOX after exposure of cells cultivated in 3D embedded in collagen I and exposure to the drugs for 30 min. (**E**) EC_50_ values for 4T1 cells overexpressing hLOX or hLOXL2 cultivated in 2D and 3D culture and exposure to DOX for 30 min. Error bars: ±SEM, n = 4.

The impaired diffusion observed in the spheroid assays should also reduce the efficacy of drugs to act as cytotoxic agents. Evidently, a possible effect of matrix crosslinking on cytotoxic activity should only be observable in a 3D setting, when cells are surrounded by a matrix that can act as a protective barrier. Furthermore, protection from exposure to drugs by impaired diffusion through a highly cross-linked matrix should not be a factor when tumor cells are exposed to the agents over several days—a standard practice for testing the cytotoxic potential of drugs *in vitro*^39^,^40^. Thus, to test the potential effect of a physical barrier function of the ECM on drug efficacy, 3D-embedded cells have to be exposed to tested drugs only for a short period of time. Limiting the time that drugs are allowed to reach the tumor cells by diffusion increases the impeding effect of a physical diffusion barrier and simultaneously reflects the situation *in vivo* more closely, where small molecule drugs are cleared relatively fast from the blood stream. Based on this considerations, we evaluated cell toxicity of two standard chemotherapeutic drugs, DOX and paclitaxel (PTX), on MT6, EMT6 and 4T1 cells, in three different settings: standard long-term exposure (72 h) of cells grown in 2D, short term exposure (30 min) followed gradual removal of the drug from the media in 2D, and short term exposure under the same conditions of 3D collagen-embedded tumor cells (Fig. 5A). As the spheroid diffusion experiments indicated that lysyl oxidases only significantly affect drug diffusion at reduced pO_2_, these experiments were performed with cells cultivated at 2% O_2_ levels. When tumor cells were exposed to the two potent cytotoxic drugs DOX and PTX for 72 h (Fig. 5B), we recorded EC_50_-values in the medium nanomolar range with sensitivities varying only by factor of four (from 19.0 ± 3.6 nM for 4T1/DOX to 67.8 ± 16.2 nM for EMT6/DOX). Not surprisingly, reduction to the short exposure time significantly reduced the toxicity of the drugs in all pairings (Fig. 5C). In the short-term system, we also tested if treatment with βAPN, either by lysyl oxidase inhibition or by an alternative mechanism, has an effect on the toxicity of the chemotherapeutics. Even at a concentration of 500 μM βAPN had no toxic effects on the tested tumor cells (Supplemental Fig. 5). Moreover, it also did not affect the toxicity of DOX or PTX (Fig. 5D). Embedding and treating the tumor cells in a 3D collagen matrix further reduced cell toxicity by at least one order of magnitude (Fig. 5E). The matrix embedding similarly reduced apparent cell toxicity of both drugs. Importantly, only in the 3D setting lysyl oxidase inhibition with βAPN decreased cell toxicity of the drugs, which further validated the concept that lysyl oxidases interfere with drug efficacy by reducing matrix diffusivity of small molecule drugs.

We next tested the effect of recombinant LOX and LOXL2 overexpression on the cytotoxic potential of DOX on 4T1 cells. Overexpression of hLOX or hLOXL2 did not affect sensitivity of 4T1 cells towards DOX when grown in 2D (Fig. 5E). However, after embedding into a 3D collagen-matrix, overexpression of hLOX and hLOXL2 significantly reduced cell toxic effects of DOX on the 4T1 cells.

**Figure 6:**
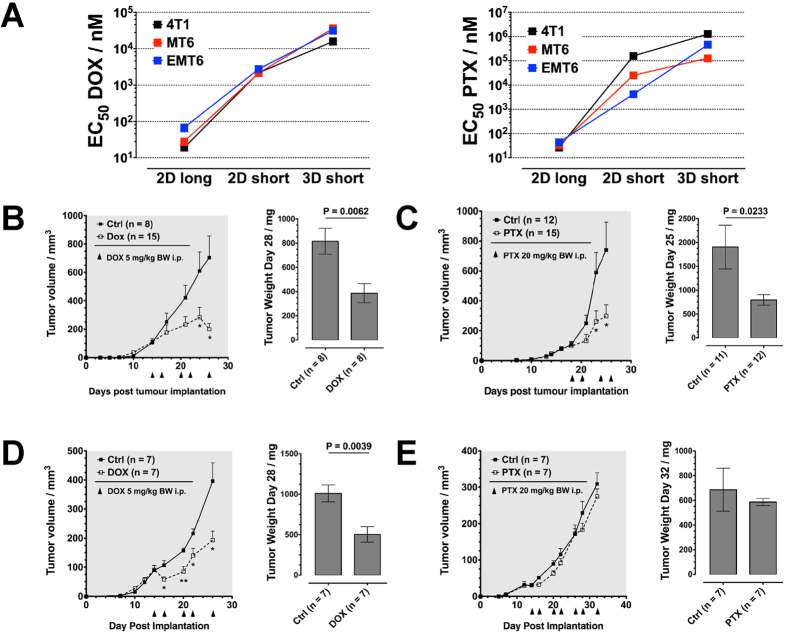
Results from 3D Cytotoxicity Assays Predict Tumor Resistance to Cytotoxic Drugs. (**A**) Dependency of drug efficacy on cultivation and exposure conditions. Effect of (**B**) DOX and (**C**) PTX treatment on MT6 tumors. Fully established tumors were treated at the indicated days with DOX (5 mg/kg BW) or PTX (20 mg/kg BW). Displayed are tumor volume measured at indicated days by caliper and mass of dissected tumors. Effect of (**D**) DOX and (**E**) PTX treatment on 4T1 tumors. Error bars: ±SEM. *indicates statistical significance versus control, *P < 0.05, **P < 0.01.

An interesting finding of the *in vitro* studies was, that while the efficacy of the chemotherapeutics was gradually reduced when cells were shorter exposed to the drugs and better protected by embedding into a collagen matrix, the extent of this effect was drastically different for the various cell/line drug parings (Fig. 6A,B). Most remarkably, 4T1 cells, which were equally sensitive to PTX and DOX in long-term exposure experiments (EC_50_ = 26.4 ± 5.4 nM and 19.0 ± 3.6 nM, respectively), were much less affected in short-term exposure experiments by PTX, with EC_50_-values differing by two orders of magnitude from the sensitivity towards DOX (160 ± 10.6 μM in 2D, 1280 ± 40.2 μM in 3D for PTX; 2.19 ± 0.46 μM in 2D, 15.9 ± 1.07 μM in 3D for DOX). Conversely, in each of the assay settings the sensitivity of MT6 cells to both tested drugs was similar. If the short-term exposure settings indeed better reflects the situation *in vivo*, 4T1 tumors should be resistant to PTX abut sensitive to DOX, while MT6 tumors should be sensitive to both drugs. To test this hypothesis we implanted mice with 4T1 and MT6 cells respectively, and treated established tumors with either of the two chemotherapeutics. Treatment was performed using established protocols (5 mg/kg BW DOX or 20 mg/kg BW PTX)^41^. The MT6 fibrosarcoma indeed reacted with significantly reduced growth to treatment with either DOX or PTX (Fig. 6B,C), reflecting the similar sensitivity of the cells *in vitro*. Conversely, 4T1 tumors responded well to DOX with strongly reduced growth, but PTX at the given dosage was not able to affect a significant reduction in tumor growth (Fig. 6D,E). Thus, from the results of the 3D cell toxicity experiments we were able to predict the insensitivity of 4T1 tumors towards PTX. Importantly, this resistance would not have been predictable from *in vitro* assays under standard long-term exposure conditions.

## Discussion

In this study we have demonstrated that lysyl oxidase activity affects drug efficacy in a direct way by increasing the barrier function of the ECM even for small molecule drugs. The results indicate that interfering with this process by inhibiting lysyl oxidase activity has the potential to increase the efficacy of systemic treatment with various drugs.

Central to our approach was the utilization of tumor spheroid diffusion assays. The spheroids allow growing cells in a compact 3D environment with tightly packed cells resembling the situation of tumor tissue. It has already been shown that tumor spheroids reflect many aspects of tumor tissue more closely than cells grown in 2D^32^,^42^,^43^,^44^,^45^. Transferring tumor cells in a 3D setting substantially alters their metabolism, proliferation rate and expression profile. These changes also affect drug sensitivity, and tumor cells are substantially more resistant to drugs in a 3D environment than in 2D culture. Imamura *et al.* have recently demonstrated that the reduced drug sensitivity in the 3D environment results from a lower proliferation rate and reduced expression of caspase-3, which is involved in the apoptotic cascade^42^. Also the impaired oxygenation in tumor spheroids leads to up-regulation of Hif1α and subsequently to increased expression of ABC transporters like MDR1^43^. These studies focused on the changes in tumor cell behavior in the 3D environment. However, an important aspect of 3D-cultures is the ECM itself, whether supplied as an embedding media or directly produced by the cells as an anchoring matrix. In addition to previous work our study demonstrate the protective effect of the matrix as a physical barrier that impedes drug transport to the cells. Direct effects of lysyl oxidases on drug sensitivity can be excluded as inhibition with βAPN in our experiments only increased efficacy of the tested chemotherapeutics when cells were embedded in a collagen matrix. Without a surrounding modifiable matrix lysyl oxidase inhibition did neither influence cell proliferation by itself nor the sensitivity towards drugs. Equally, we were able to show that overexpression of hLOX or hLOXL2 did not alter the sensitivity to cytotoxic drugs, but protected the cells from exposure to the agents by modifying the surrounding matrix. Consequently, inhibition of lysyl oxidases increased the amount of drugs reaching the cells, significantly reducing the concentration needed to cause a cytotoxic effect. Improving drug distribution and cytotoxicity by lysyl oxidase inhibition was successful in multiple genetically different cell lines, which also originated in different organs, an important result as the heterogeneity between tumors often precludes successful implementation of a therapeutic strategies over a range of different tumors. The tested cell lines showed very different patterns of expression and induction by hypoxia of the LOX family members. That treatment with βAPN was able to significantly improved delivery and response in these diverse cell lines further underlines the functional redundancy of the LOX family members and simultaneously reveals the clinical potential of a potent pan-lysyl oxidase inhibitor as an auxiliary agent to improve treatment efficacy. βAPN itself has unfavorable pharmacological properties and is an only moderately active inhibitor, however due to the discovery that the lysyl oxidases are essential in the formation of a malignant tumor microenvironment several more promising inhibitors are currently under development.

It is important to realize that the different aspects that contribute to reduced drug sensitivity in both 3D culture and *in vivo* are interdependent. Effects that impair drug diffusion also reduce transport of oxygen. Previous studies showed that apparent drug diffusion constant and pO2 in tumors is correlated^46^,^47^. Subsequently, increased lysyl oxidase expression should lead to increased hypoxia. Hypoxic tissue is less sensitive to cytotoxic therapeutics. As mentioned above, one reason is the up-regulation of efflux pumps, as ABC transporters like MDR1 and BCRP that are positively regulated by Hif1α^43^,^48^,^49^. Hence, it is possible that treatment with βAPN in our experiments in addition to evidently improving drug diffusion also enhanced drug efficacy and drug accumulation in the spheroids’ cells by increasing oxygen supply and subsequent down-regulating ABC transporters. This hypothesis needs to be examined further in subsequent studies.

We only could detect a significant dependency of drug diffusion from lysyl oxidase expression in tumor spheroids at a reduced oxygen level of 2%. At this reduced O_2_ concentration lysyl oxidases activity was significantly up-regulated in all cell lines. Which of the five lysyl oxidase family members was up-regulated at reduced pO_2_ differed between the tested cell lines, confirming previously published results^36^. Importantly, cultivation of cells at 2% O_2_ mirrors the actual oxygenation status of solid tumors. In tumors pO_2_ ranges between 2.5 and 15 mmHg (0.5–2.0 × 10^3^ Pa, or 0.5–2.0 × 10^−2^ Atm) and in normal organs between 15 and 75 mmHg^46^,^50^. In cell culture media pO_2_ levels in equilibrium are around 144 mmHg under standard conditions (19% O_2_, 80% N_2_, 5% CO_2_, 5.5% H_2_O), considerably higher than even in healthy well-perfused tissue. Thus, reduction to 2% O_2_ (pO_2_ = 15.2 mmHg) in a suitable incubator, reflects better the conditions within a still comparatively well-oxygenated tumor. Thus, the often-used terms “hypoxic conditions” for cell cultivation at less than 5% O_2_ and “normoxic conditions” for cell cultivation at 20% O_2_ are misleading. Indeed cultivation at 20% O_2_ subjects the cells to considerable oxidative stress and reflects the situation neither in a poorly supplied tumor nor in normal tissue.

In addition to oxygen concentration the outcome of the drug sensitivity assays relied strongly on the treatment time with the cytotoxic agents. While it is not only intuitive but long established that reducing the time that cells are subjected to a treatment decreases its effect^51^, both screening for new anti-neoplastic drugs and pharmacological characterization of tumor cell lines is still often performed by prolonged exposure of tumor cells to the test compounds. An exposure time of 72 h—which we also used in our experiments—appears to be established as the standard time frame for these experiments^39^,^40^. However, in our experiments the relative sensitivity of the tumor cells towards the tested chemotherapeutics after such a prolonged exposure did not reflect at all the situation we later observed *in vivo*. Small molecule drugs have only a short half-life in the circulation, before being removed from the blood stream by hepatic metabolism or renal clearance. After i.v. administration doxorubicin reaches were rapidly a peak concentration, which is also fast reduced by biliary excretion. The AUC_(0→20 min)_ contributes up to 50% of the AUC, thus the main toxicity of the drug is caused within the very early phase after administration^52^. In our *in vitro* experiments we tried to emulate the short exposure time to drugs *in vivo*. As anticipated, this significantly increased the concentration necessary to cause toxicity. Reduction of exposure times affected EC_50_ levels for PTX stronger than those for DOX. This also could be expected, because PTX exhibits a cell-cycle specific activity, being most effective against cells in G2-M transition, thus being effective only to a subset of tumor cells at a given time point^53^. However, surprisingly the increase did not run in parallel for all drug/tumor cell line combinations. PTX displayed a very similar toxicity in all tested cell lines when the cells were exposed to it for a prolonged time (72 h). At shorter exposure times EC_50_ levels differed substantially between the cell lines. In particular 4T1 cells appeared in long term exposure experiments as sensitive to PTX as the other tested cell lines, and their relative resistance was only evident in short term exposure experiments. We were able to corroborate the differences in sensitivity towards PTX that is apparent only in the short-term exposure experiment *in vivo*. These findings challenge strongly the idea that the results obtained in standard screening processes that measure pharmacological effects after prolonged exposure are even useful for side-by-side comparisons, as the *in vivo* efficacy of the tested substances can no longer be simply extrapolated from the *in vitro* results. However, a simple change of the time that cells are subjected to the tested drugs could result in more meaningful results from *in vitro* assays.

In the last years the role of lysyl oxidases in enhancing tumor angiogenesis, invasiveness and metastasis, have been extensively examined and is meanwhile established^17^,^28^,^29^,^34^,^35^,^54^. Together with our results this indicates that a potent lysyl oxidase inhibitor or therapeutic modulation of lysyl oxidase activity could serve a double purpose in the management of cancer, by simultaneously reducing malignancy of tumors and improving sensitivity towards concomitant treatment with anti-neoplastic drugs.

## Methods

If not otherwise indicated, chemicals were purchased from Sigma Aldrich (Munich, Germany) or Carl Roth (Karlsruhe, Germany).

### Cell culture

MT6 (CRL-2805), 4T1 (CRL-2539), LLC (CRL-1642) cells were obtained from ATCC (Manassas, VA). EMT6 cells have been purchased from NCI Tumor Repository (http://ncifrederick.cancer.gov/Services/NcifRepositories.aspx). All tumor cells were maintained in DMEM (Gibco, Lifetechnologies) with Penicillin/Streptomycin at 37 °C, 5% CO_2_. Cell lines were tested for mycoplasma contamination.

### D-Cell Spheroid drug penetration assay

Tumor spheroids were generated by the liquid overlay technique, using the protocol from Walser *et al.* with slight modifications^31^. Wells of a 96-well culture plate were coated with 45 μL of 1% agarose. After the agarose had solidified, 1000 tumor cells were seeded in 200 μL medium (supplemented with 500 μM βAPN were applicable) on top of the coating. Cells were incubated at 37 °C, 5% CO_2_ for six days. To test the effect of hypoxia, the forming spheroids were transferred after 96 h to an incubator with oxygen levels set to 2%. Spheroids were transferred to an 8 well cover slip slide (μ-slide, Ibidi, Martinsried, Germany) in 180 μL of medium. 60 μL of 40 μg/mL doxorubicin hydrochloride in PBS were added, and drug penetration was measured using an inverted laser-scanning confocal microscopy (Nikon A1, 20× objective), taking images at 30 min time intervals for four hours. 12 spheroids/assay condition were measured in parallel. The focal plane was identical for spheres. To ensure viability throughout the spheroid in the focal plane, the gfp signal was also recorded.

Acquired images were evaluated using the software ImageJ (http://imagej.nih.gov/ij/). All images of spheroids in a single experiment were transformed into binary images using the same threshold. Average penetration depth was calculated from the doxorubicin stained area and the total area of the spheroids.

### Cell toxicity studies

For 2D studies cells were seeded into 96-well dishes at 2500 cells/well in 150 μL full media (supplemented with 500 μM βAPN were applicable) and incubated at 37 °C, 5% CO_2_, 2% O_2_. After 48 h 50 μL of DMEM containing four times the indicated concentration of cytotoxic chemotherapeutics were added to each well without prior removal of medium. Each concentration was tested in a 6-fold replicate. Cells were incubated with the therapeutics for 72 h before media was removed. To study the long-term effect of the therapeutics, cells were incubated with the therapeutics for 72 h before media were removed. For short-term exposure, media was removed after 60 min and cells were carefully washed with three changes of media for 30 min each. Afterwards cells were incubated for 72 h.

For 3D studies, tumor cells were suspended at 1.25 × 10^5^ cells/mL in 60% (v/v) full medium and 40% (v/v) of bovine collagen I solution (Purecol 3 mg/mL, Advanced Biomatrix, Carlsbad, CA) were added. pH was adjusted with 0.1 M NaOH (10 μL/mL suspension) and NaHCO_3_ (5 μL/mL suspension). 40 μL suspension/well were transferred to a 96-well culture plate and incubated for 60 min at 37 °C, 5% CO_2_ to let the collagen set. 160 μL full medium were added (supplemented with 625 μM βAPN were applicable) and cells were incubated at 37 °C, 5% CO_2_, 2% O_2_. After 48 h 50 μL of DMEM containing five times the indicated concentration of cytotoxic chemotherapeutics were added to each well without prior removal of medium. Each concentration was tested in a 6-fold replicate. Media was removed after 60 min and cells were carefully washed with three changes of media for 30 min each. Afterwards embedded cells were incubated for 72 h.

Relative remaining viable cells were determined using the resazurin assay. EC_50_-values were determined by using a non-linear regression based model using the Prism software (Prism 5, GraphPad, LaJolla, CA).

### Lysyl oxidase activity assay

A modified form of the fluorometric microwell assay described by Fogelgren was used^55^. In short: In a black 96-well microtiterplate was added to 120 μL of an freshly prepared assay solution consisting of 75 μL 2× PBS, 15 μL N-Acetyl-Resorufin (100 μM, Ampliflu-Red, Sigma-Aldrich), 15 μL 2,5-diaminopentane (100 mM, cadaverine a substrate of all five lysyl oxidases^55^,^56^,^57^) and 15 μL horseradish-peroxidase (5 U/mL, Sigma-Aldrich). For each sample the assay solution was prepared in two triplicate rows. To one triplicate 30 μL water, to the other 30 μL βAPN (3.5 mM in water) was added. Finally 50 μL of cell culture supernatant or protein containing lysate was added and the fluorescence of released resorufin was recorded continuously over 3 h in a fluorescence plate reader (PerkinElmer, Wallac II; Ex: 530 nm, Em: 570 nm). Slope of the fluorescence signal in the linear range was calculated for both βAPN-inhibited and non-inhibited samples; the difference was used as a measure of lysyl oxidase activity.

### Promotor Analysis

Sequences were retrieved from Ensembl (http://www.ensembl.org), including the 1500 bp 5′-flanking region and the 900 bp downstream of the transcription start point. HRE consensus sequences (5′-RCGTG-3′)^36^ were located using JASPER (http://jaspar.genereg.net/)^58^,^59^.

### mRNA expression analysis

Tumor cells were seeded at 10^4^ cells/well in 48 well plates in DMEM with 10% FBS. The cells were cultivated at either 20% or 2% oxygen (5% CO_2_) for 48 h and harvested by passive lysis. RNA was isolated using the PeqGold RNA kit (Peqlab, Erlangen, Germany) according to the manufactures instructions.

mRNA-Expression was quantified by qRT-PCR on an 7900HT Thermal cycler (Applied Biosystems, Darmstadt, Germany) using SYBR green mix (Fermentas, Darmstadt, Germany). Sequences of primers used in qRT-PCR are given in supplemental table 1. All primer pairs were designed to span at least one intron to avoid amplification of contaminating gDNA. Expression levels of lox-family members were normalized against expression of *rsp29*^60^.

### Production of lentivirial particles and generation of stable cell lines expressing hLOX and hLOXL2

The entire CDS of both hLOX and hLOXL2 CDS including the signal peptide was amplified from HUVEC cDNA. The amplified DNA was cloned behind the IRES sequence into the lentiviral vector pLVX-luc-IRES-puro (Clontech, Mountain View, CA). Lentiviral particles were generated in HEK 293T cells by co-transfection with the pCMV-dR8.9 and pCMV-VSV-G^61^ (both plasmids were obtained from Addgene, Cambridge, MA), using a standard CaCl_2_-based transfection method. Supernatant was used to transfect 4T1 tumor cells. Stable cells selected with puromycin (5 μg/mL). To generate a control cell line, 4T1 cells were thransfected with lentiviral particles produced in HEK 293T cells using the pLVX-luc-IRES-puro plasmid.

### Tumor models and treatment

All experiments involving animals were reviewed and approved by the Regional Administration of Unterfranken, Würzburg, and were performed in accordance with relevant guidelines and regulations.

#### Tumor engraftment

MT6 (10^6^ cells in PBS) fibrosarcomas were generated by subcutaneous injection in the dorsal region of female C57Bl/6J mice. 4T1 (10^5^ cells in PBS) breast adenocarcinomas were generated by injection of cells into the inguinal mammary fat pad of female Balb/c mice. Balb/c mice were purchased from Charles River, Sulzfeld Germany, Balb/c and C57Bl/6J from Jackson Labs, Bar Harbor, ME.

All animals in the individual experiments were of the same age and sex. For each experiment tumor bearing mice were randomly assigned to the different treatment groups just prior to the start of treatment. In treatment studies were tumor growth was a critical outcome assessment of tumor size was performed blinded by a second researcher.

#### Exclusion of data

Animals that never developed tumors due to take rate lower than 100% were excluded from the studies. All data from animals that died or had to be sacrificed prior to the scheduled termination of the experiment was excluded.

#### Tumor treatment

Treatment of fully established tumors started on day 12 post implantation. Doxorubicin (5 mg/kg BW) was administered by intra peritoneal injection on indicated days. Control substance for doxorubicin was 0.9% NaCl. Paclitaxel (20 mg/kg BW) was diluted in 0.9% NaCl to a final volume of 200 μL from a stock solution (6 mg/mL PTX in cremophor/EtOH 1:1) and administered by intra peritoneal injection on indicated days. Control substance for PTX was 0.9% NaCl.

3-aminopropionitrile fumarate was administered at 100 mg/kg or 30 mg/kg BW in 0.9% NaCl by daily intra peritoneal injection. Control substance was a corresponding volume of cremophor/EtOH 1:1 diluted to 200 μL in 0.9% NaCl.

Tumor growth was followed by measuring perpendicular diameters of the tumors with a vernier calliper. Tumor volume was calculated using the equation V = π/6 × l × w^2^. In addition tumors were excised post mortem and weighted. Only tumors that could be excised completely without additional invaded tissue were used for weight measurements.

To monitor intra-tumoral distribution of drugs, 50 μL of Hoechst 33342 (Sigma, 20 mg/mL in 0.9% NaCl) and 100 μL of Alexa 488 or Alexa 647-labeled isolectin GS-B4 (Life Technologies, Darmstadt, Germany. 500 μg/mL in 0.9% NaCl) were injected i.v. into tumor bearing mice 20 min before sacrificing the animal. Tumors were removed and flash frozen in OCT (Sakura Finetek Torrance, CA). Tissue was cut on a cryotom to 200 μm slices and mounted on glass slides. Z-stacks were acquired by confocal imaging in the blue and near infrared channel (Nikon A1, 20 × objective). Tissue penetration was measured as the maximal distance from the vessel surface (by Alexa 647 staining) that Hoechst 33342 staining was present using ImageJ. For this purpose the acquired z-stacks were evaluated at the same tissue depth for isolated, longitudinal cut blood vessels. The maximal distance of Hoechst 33342 staining was measured perpendicular to both sides of each blood vessel, the arithmetic mean of the two values was used. Each blood vessel was evaluated at several positions. At least 5 vessels per stack, and four stacks per biological sample were evaluated.

### Collagen Crosslinking Analysis

ECM from βAPN treated and control tumors was obtained by high salt extraction of cellular components. The insoluble ECM was re-suspended in water and used to coat glass slides (angiogenesis μ-slides, Ibidi, Martinsried, Germany) at μg/well. Interferences reflection Images were acquired as z-stacks (30 slides, z-distance: 1.0 μm) on a Nikon A1 microscope in reflection mode using am 60× oil immersion objective and an 647 nm laser following a published protocol^36^. Identifiable collagen fibers in optical fields were manually counted.

## Additional Information

**How to cite this article**: Schütze, F. *et al.* Inhibition of Lysyl Oxidases Improves Drug Diffusion and Increases Efficacy of Cytotoxic Treatment in 3D Tumor Models. *Sci. Rep.* **5**, 17576; doi: 10.1038/srep17576 (2015).

## Supplementary Material

Supplementary Information
