# Supplementary material for: Inhibition of Lysyl Oxidases Improves Drug Diffusion and Increases Efficacy of Cytotoxic Treatment in 3D Tumor Models
Source: Sci Rep. 2015 Dec 1;5:17576. doi: 10.1038/srep17576 (PMC4665164; doi:10.1038/srep17576)
Supplement: Supplementary Information [file srep17576-s1.doc]

**Inhibition of Lysyl Oxidases Improves Drug Diffusion and Increases Efficacy of Cytotoxic Treatment in 3D Tumor Models**

Friedrich Schütze^1^, Florian Röhrig^1^, Sandra Vorlová^2^, Sabine Gätzner^3^, Anja Kuhn^1^, Süleyman Ergün^1^, Erik Henke^1, 4*^

^1^ Institute of Anatomy and Cell Biology II, Universität Würzburg

^2^ Institute of Clinical Biochemistry and Pathobiochemistry, Universitätsklinikum Würzburg

^3^ Institute of Tissue Engineering, Universität Würzburg

^4^ Graduate School for Life Science, Universität Würzburg

* To whom correspondence should be addressed:

Erik Henke, PhD, Institute for Anatomy and Cell Biology**,** Universität Würzburg

Koellikerstrasse 6, D-97070 Würzburg, Germany, e-mail: [erik.henke@uni-würzburg.de](mailto:erik.henke@uni-w¸rzburg.de)

Tel: ++49-931-31-83270, Fax: ++49-931-32-93630

**Supplementary Information**


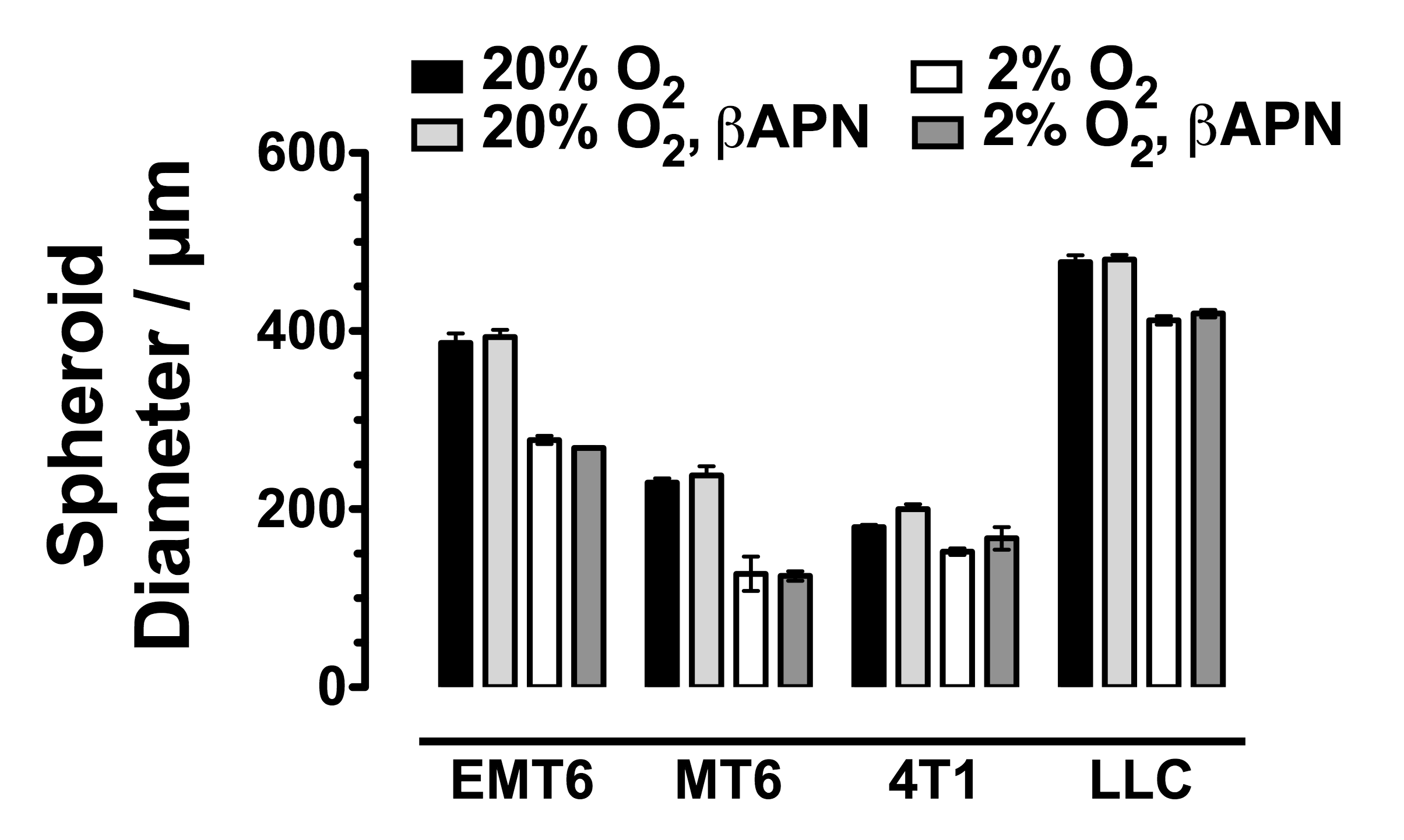
**Supplemental Figure 1: Spheroid Growth under BAPN treatment.** Spheroid diameters after 6 days of cultivation under 20 % or 2% oxygen, measured from phase contrast micrographs. While reduced oxygen supply resulted in smaller, more compact spheroids, lysyl oxidase inhibition did not affect spheroid sizes. Error bars: ±SEM.

**Supplemental Figure 2: Inhibition of lysyl oxidases inhibits collagen crosslinking in experimental tumors.**

Established tumors were treated for 10 days with βAPN (100 mg/kg BW i.p., qd). ECM from treated and control tumors was isolated by high salt extraction, the ECM used to coat chambered cover slip slides (angiogenesis µ-slides). Reflected light from collagen fibrils and evaluated by interferences reflection microscopy. (Z-Projections 4 slides, z-distance 1 µm) Scale bars: 10 µm. Error bars: ±SEM, n = 3.


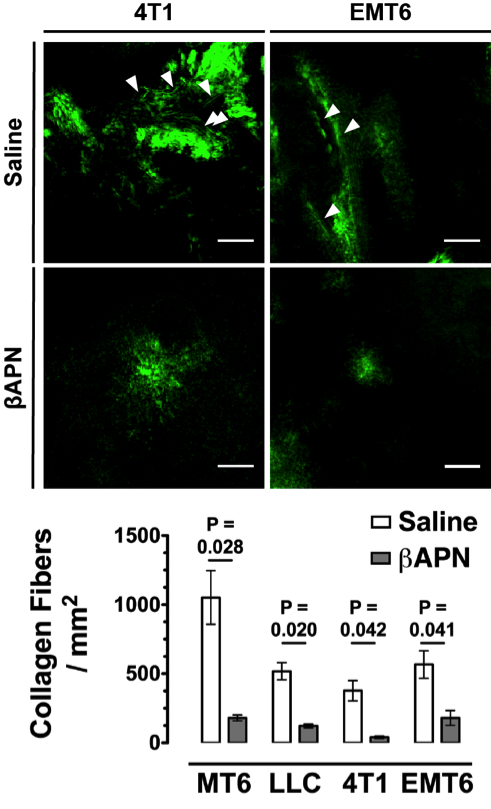


**hlox**

***-100*** GTCCGCCTTGCACGTTTCCAATCGCATTACGTGAACAAATAGCTGAGGGGCGGCCGGGCC **-40**

CAGGCGGAACGTGCAAAGGTTAGCGTAATGCACTTGTTTATCGACTCCCCGCCGGCCCGG

+215 **CTCCTTCCCTCACGTGATTTGAGCCCCGTTTTTATTTTCTGTGAGCCACGTCCTCCTCGA +275**

**GAGGAAGGGAGTGCACTAAACTCGGGGCAAAAATAAAAGACACTCGGTGCAGGAGGAGCT**

**hloxl1**

*-315* CAGAAACCATACGTGATGTCTGGGAAGTTGATCTCTCCCAGGATCTCACAAGTGCTTTTC -255

*GTCTTTGGTATGCACTACAGACCCTTCAACTAGAGAGGGTCCTAGAGTGTTCACGAAAAG*

*+830* **CCTGGTGTGGGGCGCCTGCCTGTGCGTGCTGGTGCACGGGCAGCAGGCGCAGCCCGGGCA +890**

**GGACCACACCCCGCGGACGGACACGCACGACCACGTGCCCGTCGTCCGCGTCGGGCCCGT**

**hlox4**

+ 350 TGCGCCCAGGCCTCAGGGGTGCCTGCGTGTCTTGTTTGTGTACACGCCGTGTGTAACCA +410

ACGCGGGTCCGGAGTCCCCACGGACGCACAGAACAAACACATGTGCGGCACACATTGGT

***mlox***

**+120** AGGGGCAGCGTGGAGGAGCTGTCCGCCTTGCACGTTTCC**AATCACATTACGTGAACAAAT** **+180**

TCCCCGTCGCACCTCCTCGACAGGCGGAACGTGCAAAGG**TTAGTGTAATGCACTTGTTTA**

***mloxl1***

**+230 GGCAGGCGAAGAGCCATCCTGTCCTCGAGGCCGTGGGAAGAGAAGCACGCACCGAGGCCC +280**

**CCGTCCGCTTCTCGGTAGGACAGGAGCTCCGGCACCCTTCTCTTCGTGCGTGGCTCCGGG**


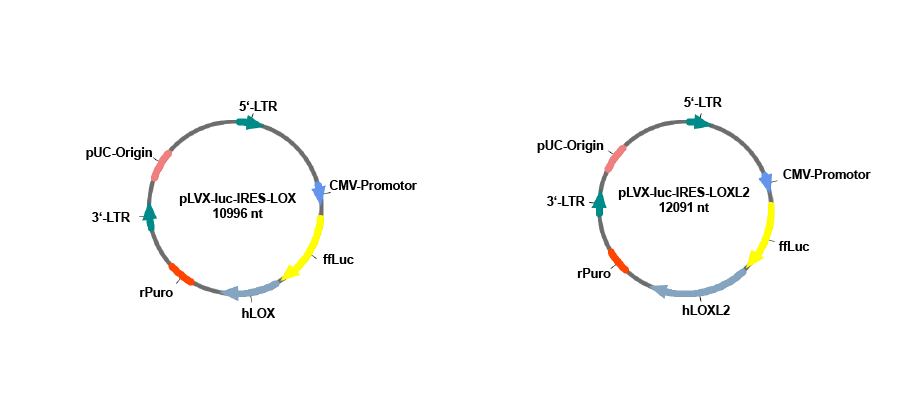
**Supplemental Figure 3: Promotor analysis for HRE.** Candidate HRE in the promotor sequences of lox(l) genes are underlined. Exon sequences are marked by red color. Position of the shown 60 bp sequences fragments relative to the transcription start site (+1) are indicated.

**Supplemental Figure 4: pLVX-DsRed–IRES-hLOX and pLVX-DsRed–IRES-hLOXL2 constructs.** Graphic representation of the pLVX-based vectors used for the generation of lentiviral particles for stable expression of hLOX and hLOXL2 in 4T1 cells.


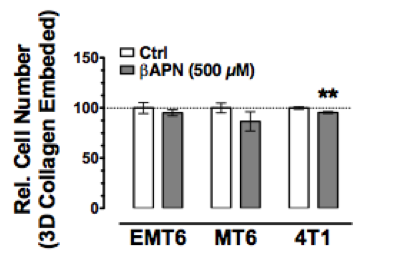

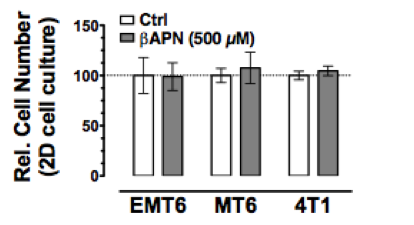


**A**

**B**

**Supplemental Figure 5: Cell Toxicity βAPN.** Normalized cell numbers after 72 h cultivation in standard growth media (DMEM, 10 % FBS) with or without supplementation of 500 µM βAPN. Cells were either grown in 2D (**A**) or in 3D matrix (**B**). Supplementation with βAPN reduced only cell growth of 4T1 cells embedded in a collagen matrix, although cell numbers were still only changed marginally (95.5 ± 2.2 % of untreated cells). Error bars: ±SEM, n = 10.

| **Supplemental Table 1 Primer sequences for RT-PCR** | | |
| --- | --- | --- |
| **Gene** | **Forward primer** | **Reverse Primer** |
| *lox* | GGCCACCCAGCCACATAGATCG | AGTAGGGGTCGGGCACCAGG |
| *loxl1* | CCGCGTGCTGGAGCCACCT | GCCTGCACGTAGTTAGGGTCCG |
| *loxl2* | TTCTTCTGGGCAACCAGGGCG | GCTAGGCTCAGGGAAGGCAGC |
| *loxl3* | TCCAGCCTCTGGAGTTGTGCC | ACGGAGACCCCACACTGAAGC |
| *loxl4* | AGGCCCGTTAGCGCTGCTCTG | TGGGGCCACATCATGGTGATTTCAG |
| *rps29* | TTCCTTTCTCCTCGTTGGGC | TCCATTCAAGGTCGCTTAGTCC |
